# Classification of patients based on their evaluation of hospital outcomes: cluster analysis following a national survey in Norway

**DOI:** 10.1186/1472-6963-13-73

**Published:** 2013-02-21

**Authors:** Oyvind Bjertnaes, Kjersti Eeg Skudal, Hilde Hestad Iversen

**Affiliations:** 1 Department for Quality Measurement and Patient Safety, Norwegian Knowledge Centre for the Health Services, Boks 7004 St Olavs plass, Oslo, 0130, Norway

## Abstract

**Background:**

A general trend towards positive patient-reported evaluations of hospitals could be taken as a sign that most patients form a homogeneous, reasonably pleased group, and consequently that there is little need for quality improvement. The objective of this study was to explore this assumption by identifying and statistically validating clusters of patients based on their evaluation of outcomes related to overall satisfaction, malpractice and benefit of treatment.

**Methods:**

Data were collected using a national patient-experience survey of 61 hospitals in the 4 health regions in Norway during spring 2011. Postal questionnaires were mailed to 23,420 patients after their discharge from hospital. Cluster analysis was performed to identify response clusters of patients, based on their responses to single items about overall patient satisfaction, benefit of treatment and perception of malpractice.

**Results:**

Cluster analysis identified six response groups, including one cluster with systematically poorer evaluation across outcomes (18.5% of patients) and one small outlier group (5.3%) with very poor scores across all outcomes. One-Way ANOVA with post-hoc tests showed that most differences between the six response groups on the three outcome items were significant. The response groups were significantly associated with nine patient-experience indicators (p < 0.001), and all groups were significantly different from each of the other groups on a majority of the patient-experience indicators. Clusters were significantly associated with age, education, self-perceived health, gender, and the degree to write open comments in the questionnaire.

**Conclusions:**

The study identified five response clusters with distinct patient-reported outcome scores, in addition to a heterogeneous outlier group with very poor scores across all outcomes. The outlier group and the cluster with systematically poorer evaluation across outcomes comprised almost one-quarter of all patients, clearly demonstrating the need to tailor quality initiatives and improve patient-perceived quality in hospitals. More research on patient clustering in patient evaluation is needed, as well as standardization of methodology to increase comparability across studies.

## Background

There is no consensus regarding how to define the quality of health care, but the patient perspective is included in many definitions [1-3]. Based on their experiences with health services, patients can evaluate the structures, processes and outcomes of care, in accordance with Donabedian’s approach to quality measurement [1]. There is a huge literature on patient evaluation of structures, processes and outcomes, and it includes various concepts such as patient satisfaction [4], patient-reported experiences [5], patient-reported outcomes [6] and health systems responsiveness [2]. These concepts are partly overlapping but also complement each other, and as a whole constitute a broad approach for the evaluation of health services from the patient perspective.

Patient satisfaction is commonly used as an outcome indicator, but tends to be highly skewed towards positive evaluations [7-10]. The same pattern can be found in customer satisfaction studies more generally [11], indicating that the measurement of satisfaction also includes sources of variation unrelated to quality. Consequently, from a quality-improvement perspective, high satisfaction scores are difficult to interpret and improve. Asking patients to report their actual experiences with health care generally results in less positive results and is easier to interpret in the clinic [12]. However, national and international patient-experience surveys show that patient-reported experiences with hospitals are also normally skewed towards positive evaluation, at least for most dimensions of care [9,10,12,13].

Several mechanisms have been used to explain this positivity bias, including sociopsychological factors such as gratitude and equity [14,15], and methodological factors such as choice of method and data-collection procedures [11,16]. This has prompted several authors to suggest that there should be a greater focus on dissatisfaction and negative evaluations [11,14]. One approach that has been used to meet this challenge is problem-oriented reporting [17], where problem scores for individual variables are reported instead of average scores with high ceiling-effects. However, the problem-oriented approach does not address whether the same patients experience problems across several variables: some patients may experience problems on many aspects, and others on some or none. Knowledge of such subgroups of patients is valuable for tailoring and implementing quality initiatives in hospitals.

Cluster analysis is a statistical technique that aims to identify homogenous groups of patients characterized by their responses to a set of variables. Cluster analysis can be used to complement the problem-oriented approach by assessing the existence and size of patient groups with systematically poorer experiences across a set of variables. These negative response groups can then be profiled by describing intra-group characteristics, and quality problems within groups might be explored to better target quality improvement initiatives. Previous patient satisfaction and experience research using cluster analysis is scarce and heterogeneous, with differences in patient groups, the statistical approach utilized and the number of response clusters [18-20], which typically ranges from two [18] to five [20]. Previous studies have also mostly been small, with questionable external validity.

In 2011, the Norwegian Knowledge Centre for the Health Services (NOKC) conducted a national patient-experience survey among hospital inpatients [21]. The questionnaire included single generic questions about outcomes related to overall patient satisfaction, malpractice and benefit of treatment. These outcome items are well suited to cluster analysis. They can be conceptually linked to the core components of quality in the Organization for Economic Cooperation and Development quality indicator project involving patient safety, responsiveness and effectiveness [3]. Furthermore, they are relevant to most hospital inpatients and are perceived as being very important by Norwegian patients [22].

The objective of this study was to identify and statistically validate clusters of patients based on their evaluation of outcomes related to overall satisfaction, malpractice and benefit of treatment. Based on the distribution characteristics of customer satisfaction [11] and previous research [18-20], we expected to identify at least two response clusters: one large group with high scores across outcomes, and one or more groups with lower scores across outcomes. We also expected the clusters to represent systematically different patient-reported experiences, since structures, processes and outcomes should be related according to Donabedian’s model [1]. Finally, case-mix studies show that individual level variables are associated with patient evaluation [23,24], and we therefore expected the most important predictors to be associated with the response clusters.

## Methods

### Data collection

The national survey included adult (16 or older) inpatients discharged from Norwegian hospitals between 1 March and 22 May 2011. A random sample of 400 patients was selected from each of the 61 hospitals, or included all eligible patients during the sampling period if the number of patients was less than 400. Additional file 1 Power calculation for the national survey was conducted at the hospital level, resulting in an appropriate sample size of 400 for each hospital. This study used data from the national patient experience survey, and the sample sizes were given by the national survey. Psychiatric units, paediatric departments and children treated at adult departments were excluded from the survey.

**Figure 1:**
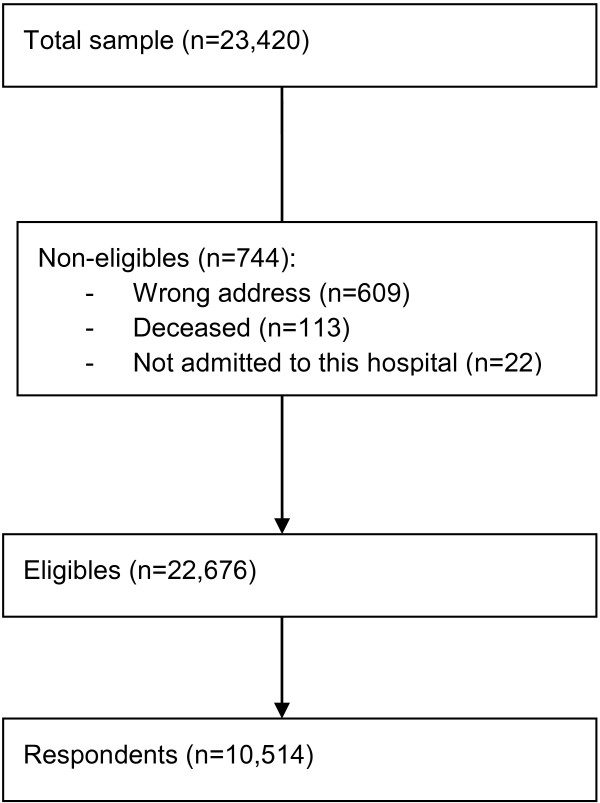
Survey flowchart.

Non-respondents were sent up to two postal reminders, the first after 3 weeks and the second a few weeks later. In total, 23,420 patients were included in the study; 744 patients were not eligible (Figure 1). All hospitals transferred data about the included patients—including age, gender, admission type, length of stay and diagnosis—to the NOKC. The Data Inspectorate and the Norwegian Ministry of Health and Care Services approved the survey.

### Questionnaire

The patient-experience questions were based on the Patient Experiences Questionnaire [25] with the response scale changed to improve the data quality [26]. The questionnaire comprised 73 closed-ended items, in addition to an open-ended question on the last page probing comments about their hospital stay or the questionnaire. Most experience items had a 5-point response format ranging from “not at all” to “to a very large extent”. Most questions are relevant for most patients. However, some questions are only relevant for a sub-group of patients, for instance cooperation between the hospital and primary health care services. These questions include a “Do not apply” category, which means that the response n for these questions and the scales they contribute to is lower than for other questions. Thirty-five items related to patient experiences with structures, processes and outcomes of health care were aggregated to 10 quality indicators in the national report, and there was good evidence for their reliability and validity [21]: waiting time (1 item), standard (6 items), next of kin (2 items), organization (4 items), doctor services (7 items), nursing services (7 items), information (3 items), discharge planning (2 items), cooperation with other health services (2 items) and patient safety (1 item). Quality indicator scores were transformed linearly to a scale of 0–100, where 100 is the best possible rating. For indicators represented by one 5-point item the following transformation was conducted: 1 = 0; 2 = 25; 3 = 50; 4 = 75; 5 = 100. For multi-item indicators patients were excluded if they responded to less than half of the items in the indicator. The internal consistency reliability of multi-item scales varied from 0.77 (cooperation with other health services) to 0.93 (doctor services). Test-retest reliability for indicators measured with single items was 0.64 (patient safety) and 0.74 (waiting time).

The patient-safety item about patient-perceived malpractice was used as one of three outcome variables in this study. Overall satisfaction and benefit of treatment were the other two outcome items. The overall patient-satisfaction question was “All in all, were the care and treatment you received at the hospital satisfactory?”, with a 5-point response format ranging from “not at all” to “to a very large extent”. The benefit-of-treatment item was “What was the overall benefit of your treatment at the hospital?”, with a 5-point response format ranging from “no benefit” to “very large benefit”. The three outcome items were included in the cluster analysis, while the nine remaining patient-based quality indicators were used to validate the cluster solution (see below).

### Statistical analysis

Descriptive data (numbers, percentages, and means) were estimated for all outcome variables and patient-experience indicators at the national level. The SPSS TwoStep Cluster Analysis procedure was used to form clusters. This procedure is well suited to large data sets and combinations of categorical and continuous variables. The three outcome variables related to overall satisfaction, malpractice and benefit of treatment were used to classify patients. Distance measures were calculated using log-likelihood, variables were standardized and autoclustering was conducted using the Schwarz Bayesian criterion. Outliers might substantially impact the outcome of hierarchical cluster analysis [27], so it was decided to create a separate cluster for outliers. Descriptive statistics were given for clusters (numbers, means, and standard deviations). One-Way ANOVA with post-hoc tests were conducted to assess differences between clusters on the three outcome items.

Validity checks are important in cluster analysis [27], and in this study involved assessing the association between clusters and the nine patient-reported experience indicators, and with sociodemographic variables known to be related to patient evaluation. The latter was achieved using one-way ANOVA for continuous variables, with Bonferroni correction for multiple comparisons, and the chi-square test for categorical variables. The second validity test involved a new cluster analysis of a random subsample of patients (50%) from the original dataset. Finally, we checked whether the degree to which comments were written in response to the open-ended question on the last page of the questionnaire differed significantly between the response clusters. Qualitative analysis reveals more critical evaluations of health-care services than does quantitative analysis [16]. Therefore, patients would be expected to write more open, qualitative comments in the most critical clusters than in other clusters. All statistical analyses were carried out using SPSS version 15.0.

## Results

The questionnaire was answered by 10,514 patients (response rate: 46.4%). Respondents were on average 61 years old (SD: 17.9), 55.1% were women, and 12.6% reported their health to be poor. 27.3% had primary school as the highest education, while 10.6% had 4 years or more of university education (Table 1). The three outcome variables were skewed towards positive evaluation: the overall score for the patient-satisfaction item was 4.2 (on a scale of 1–5, where 5 represents the best score), while those for the malpractice and benefit of treatment items were 4.6 and 4.0, respectively. Most patient-reported experience scales were also positively skewed (on a scale 0–100, where 100 represents the best score), ranging from 58 (discharge information) to 78 (next of kin).

**Table 1:** Univariate results: outcome items, patient-reported experiences and sociodemographic variables

	***n***	**%**	**Mean (SD)**
*Overall satisfaction*^a^			4.2 (0.77)
Not at all	93	0.9	-
To a small extent	170	1.7	-
To some extent	955	9.5	-
To a large extent	4797	48.0	-
To a very large extent	3987	39.9	-
*Benefit of treatment*^a^			4.0 (0.92)
No benefit	189	1.9	-
Little benefit	424	4.3	-
Some benefit	1861	18.8	-
Large benefit	4368	44.1	-
Very large benefit	3064	30.9	-
*Malpractice*^a^			4.6 (0.86)
To a very large extent	182	1.8	-
To a large extent	235	2.3	-
To some extent	653	6.4	-
To a small extent	1018	10.0	-
Not at all	8051	79.4	-
*Patient-reported experiences*^b^			
Waiting time (elective patients)	4535	-	64.0 (28.8)
Doctor services	10,153	-	73.7 (19.0)
Nursing services	10,245	-	75.4 (17.4)
Information	10,124	-	71.0 (21.0)
Contact with next of kin	7138	-	77.7 (20.8)
Standard	10,199	-	72.6 (17.2)
Organization	10,034	-	68.0 (20.2)
Discharge information	8114	-	58.3 (31.3)
Cooperation with other health services	6071	-	63.8 (29.8)
*Sociodemographic variables*			
Age, years	10,477		61.1 (17.9)
Gender, % women	10,477	55.1	-
Self-perceived health			
Excellent	693	7.5	-
Very good	1,755	19.0	-
Good	3,139	34.0	-
Rather good	2,477	26.8	-
Poor	1,162	12.6	-
Education			
Primary school	2,504	27.3	-
Secondary school	3,560	38.8	-
University/college < 4 years	2,149	23.4	-
University/college 4 years or more	974	10.6	-

^a^Scored on a scale of 1–5, where 5 represents the best score.

^b^Scored on a scale of 0–100, where 100 represents the best score.

Cluster analysis identified five response clusters, as well as a group of outliers (Table 2). Cluster 1, labelled “Excellent services”, comprised 23.5% of the patients and had close to the top score for all three outcome items. Cluster 2, labelled “Very good services, but not totally satisfied”, comprised 7.2% of the patients and resembled cluster 1, but scored significantly lower than average on satisfaction. Cluster 3, labelled “Very good services, but not totally beneficial”, comprised 15.6% of the patients, and while it also resembled cluster 1, it scored significantly lower than average for the benefit of treatment. Cluster 4, labelled “Good services”, comprised 30.0% of the patients and had average scores for the three outcome items. The fifth cluster, labelled “Services have clear improvement needs”, comprised 18.5% of the patients and had significantly lower-than-average scores for all outcome items. Cluster analysis also revealed a group of outliers who perceived services as being very poor on all outcome items (5.3% of the patients), but this group was too heterogeneous to form a cluster. One-Way ANOVA with post-hoc tests showed that the differences between clusters on the three outcome items mostly were significant, varying from 11 of 15 significant differences for group 2 to 14 of 15 significant differences for the outlier group and cluster 5.

**Table 2:** Response clusters based on patient evaluation of outcomes related to satisfaction, malpractice and benefit of treatment

	***n***	**%**	***Outcome items—centroids***^**a**^		
***Response cluster***			**General satisfaction, mean (SD)**	**Malpractice, mean (SD)**	**Benefit of treatment, mean (SD)**
Cluster 1: “Excellent services”	2241	23.5	5.0 (0.00)	4.9 (0.29)	5.0 (0.00)
Cluster 2: “Very good services, but not totally satisfied”	687	7.2	4.0 (0.20)	4.8 (0.49)	5.0 (0.00)
Cluster 3: “Very good services, but not totally beneficial”	1487	15.6	5.0 (0.00)	4.9 (0.39)	3.8 (0.36)
Cluster 4: “Good services”	2864	30.0	4.0 (0.25)	4.8 (0.55)	4.0 (0.00)
Cluster 5: “Services have clear improvement needs”	1769	18.5	3.7 (0.56)	4.5 (0.75)	2.8 (0.50)
Outlier group: “Very poor services”	504	5.3	2.9 (1.26)	2.0 (0.98)	2.7 (1.29)

^a^Scored on a scale of 1–5, where 5 represents the best score.

Clusters were significantly associated with all patient-experience indicators (p < 0.001; Table 3). One-Way ANOVA post-hoc tests showed that the outlier group was significantly different from other clusters on all experience indicators, except waiting time where no differences were observed with cluster 2, 4 and 5. Cluster 1 was significantly different from all other clusters for all nine patient-experience indicators. Cluster 2 was significantly different from other clusters, except on discharge information (cluster 3), cooperation with other health services (cluster 3) and waiting time (outlier group, cluster 3, 4 and 5). Cluster 3 was significantly different from all other clusters on all indicators, except on discharge information (cluster 2), cooperation with other health services (cluster 2) and waiting time (cluster 2 and 4). Cluster 4 was significantly different from all other clusters on all variables, except waiting time (outliers, cluster 2 and 3). Cluster 5 was also significantly different from all clusters on all indicators, except on waiting time (outliers, cluster 2).

**Table 3:** Patient-experience scores and soscio-demographic variables for response clusters

	**Cluster 1**	**Cluster 2**	**Cluster 3**	**Cluster 4**	**Cluster 5**	**Outlier group**	**Significance**^**a**^
*Patient experiences*^b^							
Waiting time (elective patients)	68.9	62.9	64.9	63.7	57.4	57.6	<0.001
Doctor services	88.5	77.4	81.1	70.9	60.4	47.5	<0.001
Nursing services	89.6	76.5	83.1	71.5	63.9	54.8	<0.001
Information	86.5	75.0	77.8	68.6	57.0	44.3	<0.001
Contact with next of kin	90.2	78.2	83.5	74.5	66.9	61.4	<0.001
Standard	83.3	72.1	79.0	68.7	63.7	60.5	<0.001
Organization	83.0	67.9	75.9	64.8	55.2	45.0	<0.001
Discharge information	75.7	62.4	64.5	55.7	43.4	34.1	<0.001
Cooperation with other health services	79.5	67.9	68.2	62.8	50.0	40.0	<0.001
*Other variables*							
Age, years (mean)	59.4	57.4	62.2	61.9	59.9	58.7	<0.001
Gender, % women	56.8	54.1	52.5	53.1	55.0	54.5	<0.05
Open comments, %	24.2	26.1	23.3	24.3	33.7	57.3	<0.001
Self-perceived health, %							<0.001
Excellent	15.2	11.5	6.8	4.2	3.2	3.5	
Very good	28.3	25.9	17.4	17.6	10.5	11.3	
Good	32.0	33.4	34.8	39.6	30.4	22.6	
Rather good	19.3	24.3	29.3	27.9	34.0	25.9	
Poor	5.2	4.9	11.7	10.7	21.9	36.8	
Education, %							<0.001
Primary school	26.3	22.4	32.0	27.0	26.4	28.7	
Secondary school	36.8	40.9	37.4	38.4	41.7	39.9	
University/college < 4 years	24.1	24.0	21.8	24.2	22.9	21.1	
University/college 4 years or more	12.7	12.7	8.8	10.4	9.0	10.3	

^a^One-way ANOVA for all tests, except gender, education, health and open comments (chi-square test).

^b^Scored on a scale of 0–100, where 100 represents the best score.

18 of 30 age comparisons between clusters were significant. The clearest pattern was that cluster 5 was significantly older than other clusters, while cluster 2 was significantly younger than most clusters. For self-perceived health, 28 of 30 comparisons were significant, and the clearest pattern was that outliers and cluster 5 had poorest health, while cluster 1 had best health. For education, only 6 of 30 comparisons were significant and half of these concerned cluster 3 with lower education than three other groups. Gender differences were mostly small between clusters, while cluster 5 and the outlier group wrote more open comments than the other clusters (33.7% and 57.3%, respectively).

A second cluster analysis of a random sample of cases produced four response clusters, as well as the outlier group (results not shown here). The clusters were very similar in size and content, except that clusters 2 and 4 from the initial analysis were collapsed.

## Discussion

This study identified five patient clusters related to the evaluation of hospital outcomes, as well as a group of heterogeneous extreme negative outliers. The first cluster groups all patients who attributed the highest scores for general satisfaction, absence of malpractice, and treatment benefit. The second cluster gave high scores to treatment benefit and absence of malpractice, but those patients were less satisfied overall than those in the first cluster. By contrast, the third cluster grouped those who were satisfied and did not describe malpractice, even if they gave a lower score to treatment benefit. The fourth cluster gave relatively balanced scores to each of the three indicators. The fifth cluster included patients not highly satisfied overall or not satisfied with treatment benefit, but who declared less malpractice. Finally the last group represents outliers who were not satisfied overall, declared malpractice, and little treatment benefit. The two groups with systematically poorer evaluation across outcomes comprised almost one-quarter of all patients, indicating the clear potential to improve hospital services from the patient perspective.

This study identified more clusters than previous studies. However, previous research in this field is scarce and heterogeneous, with differences in patient groups, statistical approaches and numbers of response clusters [18-20]. One previous study identified five clusters of patients [20], while other have identified two [18] and three [19]. The two-cluster study is not comparable to the current study, since it was restricted to one hospital and only included 47 patients with type 1 diabetes receiving kidney or pancreas-kidney transplant [18]. The two other studies were more comparable, but a main difference was that these studies restricted cluster variables to experiences and satisfaction. Therefore, at the conceptual level these studies differ from the current study. However, a common cluster pattern was identified across these studies: a top-score cluster, a medium cluster, and a low-score cluster. More research is needed on response clusters in patient evaluations, but it is important to standardize the methodology, especially with regard to handling of outliers and choice of cluster variables. We recommend excluding outliers from cluster formation, but including them in the interpretation of patient clusters. Furthermore, we recommend using relevance and importance for patients as the main criteria when selecting cluster variables. The cluster solution should include most patients, meaning that several of the patient-reported experience variables examined in this study were inappropriate, such as cooperation with other health services, where item non-response was high. From a research perspective, the middle clusters in our study should be further explored in future studies. However, from a quality-improvement perspective, these groups are not the most interesting since the overall outcomes are rated highly.

The outlier group scored poorly on all outcome items. The most striking feature of this group was the extent of perceived malpractice by the hospital: on average, these patients perceived themselves to have been subject to a large extent of hospital malpractice. The only reason the outlier group is not a cluster in a statistical sense, is the amount of internal variance on general satisfaction and benefit of treatment. However, from a quality improvement perspective this group is highly relevant. Efforts to identify, monitor and reduce the outlier group and cluster 5 should be a goal of the quality improvement work performed in hospitals. Further qualitative research should be conducted to explore quality problems within these groups, which will give valuable information when tailoring and implementing quality initiatives in hospitals. At the policy level, large differences in patient-perceived outcomes challenge both the goal of high quality and equal distribution of health-care quality [3]. In Norway, the cooperation reform began on 1 January 2012, with the aim of improving cooperation between primary and secondary health care. This reform clearly relates to the largest improvement areas for cluster 5, and so potential improvements following the reform should be evaluated in future research.

Cluster analysis can be criticized for being exploratory and atheoretical [27]. The existing research provided our study with little basis for building theoretical and analytical models. Several analytical strategies and approaches were tested before the final solution was reached; this solution was rigorously tested for validity, which resulted in further minor adjustments. All in all, we believe that the resulting response clusters have validity, but that the middle clusters should be further explored in future research. Another potential limitation is the use of single outcome items as cluster variables. Single items are normally less reliable than multi-item scales. Furthermore, patient-reported outcomes normally include both multiple scales and pre–post measurement [6]. However, psychometric evaluation is of less concern since we are clustering patients, not items. In addition, the use of factor scores in cluster analysis is debated, since research has shown that the most discriminatory variables are not well represented in most factor solutions [27]. The outcome items in this study were developed and tested in Norway and found to be very important for Norwegian patients [22]. Consequently, using these items as cluster variables appears to provide adequate validity. A third limitation is related to the response rate. More than half of the patients failed to respond to the survey. Findings from previous non-response research in Norwegian national surveys shows that low response rates have not caused serious bias in population estimates [28,29]. The findings from a Norwegian follow-up study involving the same hospital population as the current study showed that postal respondents and non-respondents had almost the same scores [30]. However, it is not unlikely that patients experiencing the poorest health care are underrepresented in patient experience surveys, for instance patients seriously harmed by adverse events. These patients constitute a minority of hospital patients, which implies only small effects on the overall survey estimates. However, there is a danger that the size of the outlier group and cluster 5 are underestimated. We stress the importance of allowing proxies to answer on behalf of patients not able to answer themselves, so that these patients can be adequately represented. The national survey included the opportunity for proxies to respond, but the current study did not have a particular focus on the association between patient clusters and non-response. This is a limitation of the study and an important area for future research. A final limitation is the fact that the study was restricted to identifying and statistically validating patient clusters. Consequently, substantial issues related to explaining and profiling clusters are warranted in future research. For instance, the importance of poor quality of care and disease severity for belonging to the outlier group and cluster 5 should be explored.

The purpose of national patient experience surveys is systematic measurement of patient experiences, as part of quality improvement, business control, free hospital choice and public accountability. The hospital level is the main level for the national surveys, and both case-mix adjusted comparisons of hospitals and unadjusted frequency based results for each hospital are presented in national reports and at several internet-sites. Patient clusters are highly relevant for both reporting types, and present a novel way of reporting and understanding patient evaluation of hospitals. The size of the improvement clusters (cluster 5 and the outlier group) can be computed, compared and presented at the hospital level. Research has shown that consumers have difficulties in understanding quality information [31], and that “less is more” in this respect [32]. Therefore, a percentage at the hospital level showing the size of improvement clusters seems appropriate in the context of presenting information to consumers. On the other hand, more specific results are called for when reporting information to health providers aiming to evaluate and improve the quality of care [33]. Consequently, both the size of cluster 5 and the outlier group should be included and constitute a fruitful supplement when reporting results to the responsible hospitals. The latter should also be supplemented with qualitative data to better understand the types of problems these clusters are facing, and profiling data to be able to target improvement initiatives within hospitals.

More research is needed to secure the usefulness of cluster analysis and reporting in this setting. The cluster approach should complement existing reports, and not only reproduce the same ratings of hospitals as the existing approach [17]. The statistical construction of the measure at the hospital level should also be further explored, especially how to handle case-mix adjustment. Furthermore, the appropriate method for analyzing and presenting qualitative data and profiling data for hospitals should be closely examined in future research. To assess generalizability, the cluster analysis should be reproduced in future national surveys in Norway and national hospital surveys in other countries. The latter can be easily done by including the three outcome items in this study and by applying the same statistical procedures as in the current article.

## Conclusions

While the patients overall provided positive evaluations of hospitals, distinct response clusters were identified, with large differences in the evaluations of hospital outcomes. One cluster had systematically poorer evaluation across outcomes, and together with a small group of extreme negative outliers these groups consisted of almost one-quarter of all patients. Further research is needed to explain and profile these clusters, but our preliminary interpretation is that these groups received poorer health care services than other clusters. The study clearly demonstrates the need to tailor quality initiatives and improve patient-perceived quality in hospitals. More research on patient clustering in patient evaluation is needed, as well as standardization of methodology to increase comparability across studies.

## Competing interests

The authors declare that they have no competing interests.

## Authors’ contributions

OAB planned the paper together with HII and KES, carried out the statistical analysis, and drafted the paper. HII participated in the planning process, revised the draft critically and approved the final version. KES participated in the planning process, revised the draft critically and approved the final version. All authors read and approved the final manuscript.

## Pre-publication history

The pre-publication history for this paper can be accessed here:

http://www.biomedcentral.com/1472-6963/13/73/prepub

## Supplementary Material

Additional file 1 Questionnaire used in the national survey in 2011. Click here for file
