# Supplementary material for: Classification of patients based on their evaluation of hospital outcomes: cluster analysis following a national survey in Norway
Source: BMC Health Serv Res. 2013 Feb 21;13:73. doi: 10.1186/1472-6963-13-73 (PMC3643880; doi:10.1186/1472-6963-13-73)
Supplement: Additional file 1 — Questionnaire used in the national survey in 2011. [file 1472-6963-13-73-S1.pdf]

Undersøkelse om pasienters erfaringer fra sykehusopphold

PasOpp Somatikk 2011

Vi ønsker å vite hvordan pasienter har det når de er innlagt på sykehus i Norge. Målet med undersøkelsen er å forbedre kvaliteten på tilbudet ved norske sykehus.

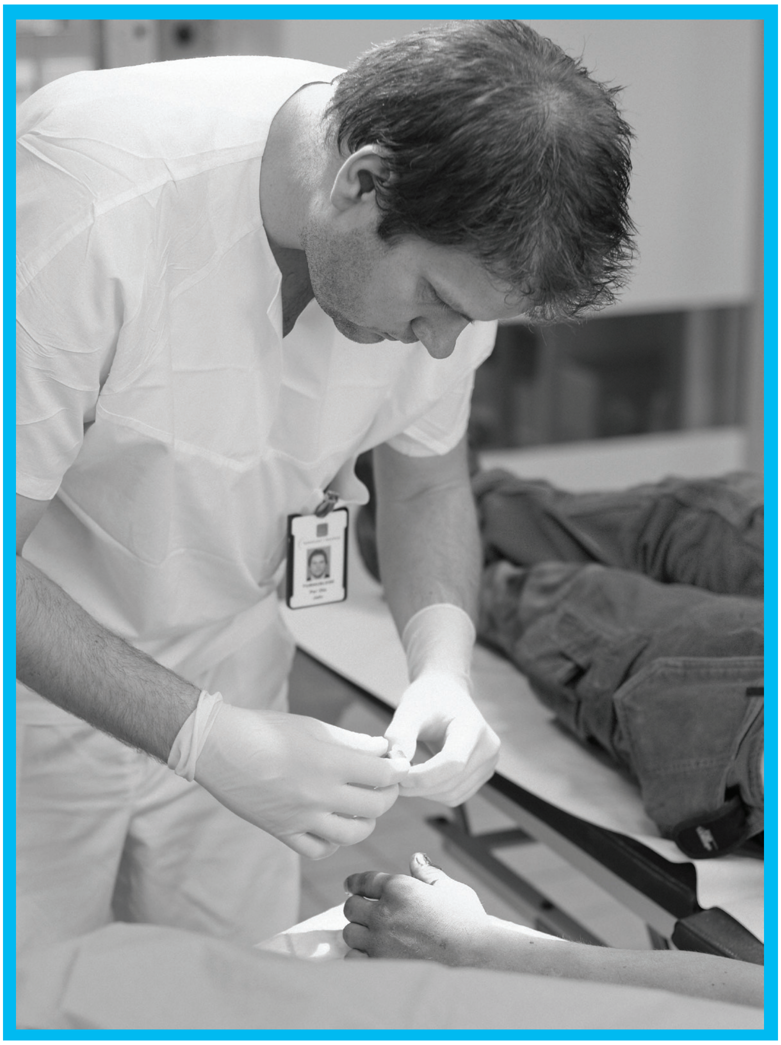

Dine erfaringer er viktige for oss og vi håper du vil bruke noen minutter på å fylle ut dette spørreskjemaet!

kunnskapssenteret
Nasjonalt kunnskapssenter for helsetjenesten

Samlet vurdering

	Ikke i det hele tatt	I liten grad	I noen grad	I stor grad	I svært stor grad
1. Var pleien og behandlingen du fikk på sykehuset, alt i alt, tilfredsstillende?	☐	☐	☐	☐	☐

	Ikke noe utbytte	Lite utbytte	En del utbytte	Stort utbytte	Svært stort utbytte
2. Hvilket utbytte har du hatt, alt i alt, av behandlingen på sykehuset?	☐	☐	☐	☐	☐

Før sykehusoppholdet

	Veldig høye	Ganske høye	Både og	Ganske lave	Veldig lave	Ikke aktuelt
3. Alt i alt, hadde du høye eller lave forventninger til sykehuset før innleggelsen?	☐	☐	☐	☐	☐	☐

	Egne erfaringer med sykehuset fra før	Andres erfaringer med sykehuset fra før	Medieoppslag om sykehuset	Medieoppslag om andre sykehus eller helsetjenester	Annet	Ikke aktuelt
4. Hva var forventningene dine basert på?	☐	☐	☐	☐	☐	☐

	Ja, altfor lenge	Ja, ganske lenge	Ja, men ikke lenge	Nei	Ikke aktuelt
5. Måtte du vente for å få et tilbud ved sykehuset?	☐	☐	☐	☐	☐

	Ja	Nei	Brukte ikke medisiner
6. Ved innleggelsen, hadde du med deg en oppdatert liste over medisiner du brukte?	☐	☐	☐

Under sykehusoppholdet: Informasjon

	Ikke i det hele tatt	I liten grad	I noen grad	I stor grad	I svært stor grad	Ikke aktuelt
7. Fikk du vite det du syntes var nødvendig om hvordan prøver og undersøkelser skulle foregå?	☐	☐	☐	☐	☐	
8. Fikk du vite det du syntes var nødvendig om resultater av prøver og undersøkelser?	☐	☐	☐	☐	☐	
9. Fikk du tilstrekkelig informasjon om din diagnose/ dine plager?	☐	☐	☐	☐	☐	
10. Fikk du vite det du syntes var nødvendig om virkninger og bivirkninger av nye medisiner du begynte med?	☐	☐	☐	☐	☐	☐

Under sykehusoppholdet: Pleiepersonalet

	Ikke i det hele tatt	I liten grad	I noen grad	I stor grad	I svært stor grad	Tilkalte ikke hjelp
11. Snakket pleiepersonalet til deg slik at du forstod dem?	☐	☐	☐	☐	☐	
12. Opplevde du at pleiepersonalet hadde omsorg for deg?	☐	☐	☐	☐	☐	
13. Har du tillit til pleiepersonalets faglige dyktighet?	☐	☐	☐	☐	☐	
14. Fikk du fortalt pleiepersonalet alt du mente var viktig om din tilstand?	☐	☐	☐	☐	☐	
15. Opplevde du at pleiepersonalet var interessert i din beskrivelse av egen situasjon?	☐	☐	☐	☐	☐	
16. Ble du tatt med på råd i spørsmål som omhandlet din pleie?	☐	☐	☐	☐	☐	
17. Hadde pleiepersonalet tid til deg når du trengte det?	☐	☐	☐	☐	☐	
18. Kom pleiepersonalet til deg så raskt som du ønsket når du tilkalte hjelp (brukte ringesnoren/ ringeklokka)?	☐	☐	☐	☐	☐	☐

Under sykehusoppholdet: Legene

	Ikke i det hele tatt	I liten grad	I noen grad	I stor grad	I svært stor grad	Ikke aktuelt
19. Snakket legene til deg slik at du forstod dem?	☐	☐	☐	☐	☐	
20. Opplevde du at legene hadde omsorg for deg?	☐	☐	☐	☐	☐	
21. Har du tillit til legenes faglige dyktighet?	☐	☐	☐	☐	☐	
22. Hadde legene tid til deg når du trengte det?	☐	☐	☐	☐	☐	
23. Fikk du fortalt legene alt du mente var viktig om din tilstand?	☐	☐	☐	☐	☐	
24. Opplevde du at legene var interessert i din beskrivelse av egen situasjon?	☐	☐	☐	☐	☐	
25. Opplevde du at behandlingen var tilpasset din situasjon?	☐	☐	☐	☐	☐	
26. Ble du tatt med på råd i spørsmål som omhandlet din behandling?	☐	☐	☐	☐	☐	☐
27. Var du involvert i avgjørelser som angikk din behandling?	☐	☐	☐	☐	☐	☐

Under sykehusoppholdet: Organisering

	Ikke i det hele tatt	I liten grad	I noen grad	I stor grad	I svært stor grad	Hadde ikke smerter
28. Opplevde du at det var en fast gruppe pleiepersonale som tok hånd om deg?	☐	☐	☐	☐	☐	
29. Opplevde du at én lege hadde hovedansvaret for deg?	☐	☐	☐	☐	☐	
30. Opplevde du at sykehusets arbeid var godt organisert?	☐	☐	☐	☐	☐	
31. Opplevde du uforutsett venting mens du var innlagt på sykehuset?	☐	☐	☐	☐	☐	
32. Opplevde du at viktig informasjon om deg hadde kommet fram til rette vedkommende?	☐	☐	☐	☐	☐	
33. Mener du at personalet gjorde alt de kunne for å gi deg effektiv smertelindring?	☐	☐	☐	☐	☐	☐

Under sykehusoppholdet: Pårørende

	Ikke i det hele tatt	I liten grad	I noen grad	I stor grad	I svært stor grad	Ikke aktuelt
34. Ble dine pårørende tatt godt imot av personalet på sykehuset?	☐	☐	☐	☐	☐	☐
35. Var det enkelt for dine pårørende å få informasjon om deg mens du var innlagt på sykehuset?	☐	☐	☐	☐	☐	☐

Standard og omgivelser

	Ikke i det hele tatt	I liten grad	I noen grad	I stor grad	I svært stor grad	Ikke aktuelt
36. Fikk du inntrykk av at sykehusets utstyr var i god stand?	☐	☐	☐	☐	☐	
37. Fikk du inntrykk av at sykehuset for øvrig var i god stand?	☐	☐	☐	☐	☐	
38. Var rommet du lå på tilfredsstillende?	☐	☐	☐	☐	☐	
39. Var muligheten for ro og hvile tilfredsstillende?	☐	☐	☐	☐	☐	
40. Var maten tilfredsstillende?	☐	☐	☐	☐	☐	
41. Var renholdet tilfredsstillende?	☐	☐	☐	☐	☐	
42. Var muligheten for samvær med besøkende tilfredsstillende?	☐	☐	☐	☐	☐	☐

Pasientsikkerhet ved sykehuset

	Ikke i det hele tatt	I liten grad	I noen grad	I stor grad	I svært stor grad
43. Mener du at du på noen måte ble feilbehandlet av sykehuset (etter det du selv kan bedømme)?	☐	☐	☐	☐	☐

	Nei	Ja, én gang	Ja, flere ganger
44. Opplevde du administrative feil under sykehusoppholdet (f.eks. rot i papirene, prøvesvar uteble, ble ikke innkalt som avtalt, avtalte undersøkelser ble ikke bestilt)?	☐	☐	☐
45. Opplevde du at personalet glemte å kontrollere identiteten din?	☐	☐	☐
46. Opplevde du at personalet glemte å gi deg viktig informasjon?	☐	☐	☐

	Ikke i det hele tatt	I liten grad	I noen grad	I stor grad	I svært stor grad	Vet ikke	Ikke aktuelt
47. Fikk du feil eller forsinket diagnose i forbindelse med sykehusoppholdet?	☐	☐	☐	☐	☐	☐	☐
48. Fikk du unødig skade eller unødig problem som følge av et kirurgisk inngrep eller en undersøkelse?	☐	☐	☐	☐	☐	☐	☐
49. Opplevde du å få feil medisiner eller på annen måte å bli feilmedisinert?	☐	☐	☐	☐	☐	☐	☐
50. Opplevde du mangelfull håndhygiene (håndvask) blant personalet?	☐	☐	☐	☐	☐	☐	☐

	Nei	Ja	Hadde infeksjon før innleggelsen
51. Fikk du infeksjon i forbindelse med sykehus-oppholdet? (f.eks. betennelse/puss i operasjonssår, lungebetennelse, blodforgiftning eller urinveisinfeksjon/blærekatarr)?	☐	☐	☐

52. Hvis du opplevde feil eller unødig problem i forbindelse med sykehusoppholdet, tok personalet hånd om feilen eller problemet på en tilfredsstillende måte?

Ikke i det hele tatt	I liten grad	I noen grad	I stor grad	I svært stor grad	Personalet visste ikke om det	Ikke aktuelt
☐	☐	☐	☐	☐	☐	☐

Under sykehusoppholdet: Forberedelse til tiden etter utskrivning

	Ikke i det hele tatt	I liten grad	I noen grad	I stor grad	I svært stor grad	Ikke aktuelt
53. Ble du informert om hva du selv kunne gjøre hjemme ved eventuelle tilbakefall?	☐	☐	☐	☐	☐	☐
54. Ble du informert om hvilke plager du kunne regne med å få i tiden etter sykehus-oppholdet?	☐	☐	☐	☐	☐	☐

	Ja	Nei	Ikke aktuelt
55. Ble en oppdatert liste over medisinene dine gjennomgått med deg da du ble utskrevet fra sykehuset?	☐	☐	☐

	Ikke i det hele tatt	I liten grad	I noen grad	I stor grad	I svært stor grad	Ikke aktuelt
56. Hadde du ubesvarte spørsmål om medisinene dine da du ble utskrevet fra sykehuset?	☐	☐	☐	☐	☐	☐

Sykehusets samarbeid med andre

	Ikke i det hele tatt	I liten grad	I noen grad	I stor grad	I svært stor grad	Vet ikke	Ikke aktuelt
57. Opplever du at sykehuset har samarbeidet bra med fastlegen din om det du var innlagt for?	☐	☐	☐	☐	☐	☐	☐
58. Opplever du at sykehuset har samarbeidet bra med hjemme-tjenesten/ andre kommunale tjenester om det du var innlagt for?	☐	☐	☐	☐	☐	☐	☐

Andre vurderinger

	Mye dårligere enn forventet	Noe dårligere enn forventet	Som forventet	Noe bedre enn forventet	Mye bedre enn forventet
59. Hvis du ser hele sykehusoppholdet under ett, hvordan vil du vurdere sykehuset?	☐	☐	☐	☐	☐

	Mye dårligere enn forventet	Noe dårligere enn forventet	Som forventet	Noe bedre enn forventet	Mye bedre enn forventet
60. Har sykehusoppholdet gjort din helsetilstand dårligere enn forventet, som forventet eller bedre enn forventet?	☐	☐	☐	☐	☐

	Ikke i det hele tatt	I liten grad	I noen grad	I stor grad	I svært stor grad
61. Ville du anbefalt dette sykehuset til familie og venner?	☐	☐	☐	☐	☐

Bakgrunnsspørsmål

62. Da du reiste fra sykehuset, reiste du til en annen helseinstitusjon eller hjem?

Annen helse-institusjon	Hjem til meg selv	Hjem til andre
☐	☐	☐

63. Stort sett, vil du si din helse er...

Utmerket	Meget god	God	Nokså god	Dårlig
☐	☐	☐	☐	☐

64. Hvor mange ganger har du vært innlagt på sykehus de siste to årene?

1 gang	2 ganger	3 - 5 ganger	6 - 10 ganger	Mer enn 10 ganger
☐	☐	☐	☐	☐

65. Hvorfor var du innlagt på sykehuset? *Du kan sette flere kryss.*

Utredning	Behandling	Oppfølging/ kontroll	Annet
☐	☐	☐	☐

66. Hvem har fylt ut spørreskjemaet?

Pasienten selv	Pårørende til pasienten
☐	☐

Spørsmålene 67 - 73 gjelder deg som har svart på spørreskjemaet

67. Er du mann eller kvinne?

Mann	Kvinne
☐	☐

68. Hva er din alder?

År
☐

69. Er du gift eller samboende?

Ja, gift	Ja, samboende	Nei	Enke/ enkemann
☐	☐	☐	☐

70. Hva er din høyeste fullførte utdanning?

Grunnskole	Videre-gående skole	Høyskole/ universitet (1-4 år)	Høyskole/ universitet (mer enn 4 år)
☐	☐	☐	☐

71. Hva gjør du til daglig? *Sett kun ett kryss.*

Yrkesaktiv	Sykmeldt, på uføretrygd eller arbeids-avklaring	Pensjonist	Under utdannning	Hjemme-arbeidende	Arbeids-ledig	Annet
☐	☐	☐	☐	☐	☐	☐

72. Hva er morsmålet ditt?

Norsk	Samisk	Annet nordisk språk	Annet europeisk språk	Ikke-europeisk språk
☐	☐	☐	☐	☐

73. Kunne du tenke deg å svare på et nytt spørreskjema fra oss om kort tid, dersom det blir aktuelt?

☐ Sett kryss her hvis du kunne tenke deg å svare på et nytt skjema

Kommentarer til sykehusoppholdet eller spørreskjemaet:

Her kan du f.eks. skrive mer om feil eller unødig problem under sykehusoppholdet, komplikasjoner etter oppholdet, eller tilsvarende problemer ved tidligere opphold ved dette sykehuset.

Vennligst ikke skriv under denne linje
